# The effect of a running task on muscle shear elastic modulus of posterior lower leg

**DOI:** 10.1186/s13047-017-0238-x

**Published:** 2017-12-12

**Authors:** Shuhei Ohya, Masatoshi Nakamura, Takafumi Aoki, Daichi Suzuki, Takanori Kikumoto, Emi Nakamura, Wataru Ito, Ryo Hirabayashi, Tomoya Takabayashi, Mutsuaki Edama

**Affiliations:** 1 Department of Physical Therapy, Niigata University of Health and Welfare, 1398 Shimami-cho, Kita-ku, Niigata City, 950-3198 Japan; 2 Institute for Human Movement and Medical Sciences, Niigata University of Health and Welfare, 1398 Shimami-cho, Kita-ku, Niigata City, 950-3198 Japan

**Keywords:** Medial tibial stress syndrome, Flexor digitorum longus, Tibialis posterior, Shear elastic modulus, Running

## Abstract

**Background:**

Medial tibial stress syndrome (MTSS) is one of the most common causes of exercise-related leg pain in runners. Because stopping training due to pain from MTSS could decrease the athlete’s competitiveness, it is necessary to construct MTSS prevention and treatment programs. However, the effect of running, which is believed to cause MTSS, on shear elastic modulus of the posterior lower leg is unclear. Therefore, the purpose of this study was to investigate the effect of 30 min of running on shear elastic modulus of the posterior lower leg in healthy subjects.

**Methods:**

Twenty healthy males volunteered to participate in this study (age, 20.9 ± 0.6 y; height, 169.6 ± 4.5 cm; weight, 62.6 ± 5.2 kg). The shear elastic modulus of the posterior lower leg was measured using ultrasonic shear wave elastography before and immediately after a 30-min running task.

**Results:**

Shear elastic moduli of the flexor digitorum longus and tibialis posterior were significantly increased after 30 min running task. However, there were no significant changes in shear elastic moduli of the lateral gastrocnemius, medial gastrocnemius, peroneus longus and peroneus brevis.

**Conclusion:**

The results suggested that the increases in shear elastic moduli of flexor digitorum longus and tibialis posterior after running could be a risk factor for running-related MTSS development.

## Background

Medial tibial stress syndrome (MTSS) is one of the most common causes of exercise-related leg pain in runners [1, 2]. MTSS was thought to occur from repetitive running on hard surfaces or forcible [3]. Previous studies have reported that MTSS accounted for 9.5% of all injuries and 60% of injuries to the lower limbs in athletes [4], and MTSS was the most frequent running disorder [5]. In addition, athletes with MTSS should stop participating in competition for up to 16 week [6], and a longer duration was necessary until the athletes with MTSS could run with a pain score of 4 or less on a 10-point scale [7]. Moreover, MTSS has a high recurrence rate [1]. Therefore, since stopping training due to pain from MTSS could decrease the athlete’s competitiveness, it is necessary to construct MTSS prevention and treatment programs. Based on the injury prevention model proposed by Van Mechelen [8], it is necessary to identify the mechanism and factors of injury to prevent the injury, and this might be useful for constructing the injury prevention protocol.

Although the mechanisms and risk factors for MTSS are not clear, the symptoms of MTSS could be due to the stress response of the fascia and periosteum at the medial border of the tibia [9]. In addition, in the cadaveric study, Edama et al. reported that flexor digitorum longus (FDL) of almost all subjects and the soleus (SOL) of approximately half of the subjects were attached to the posteromedial border of the tibia, which is the most common site affected by MTSS [10]. These results suggest that MTSS could be caused by the excessive elongational stress and muscle stiffness of the FDL and/or SOL. However, since there have been few studies of the passive tension applied to muscles and/or muscle stiffness in vivo, it is unclear if these might be involved with the mechanisms and risk factors for MTSS.

Recently, an ultrasound elastography technique, known as ultrasonic shear wave elastography (SWE), has emerged as a method for estimating shear elastic modulus [11]. Since previous studies showed that there was a significant correlation between shear elastic modulus measured by SWE and passive tension [12, 13], shear elastic modulus could be used as an indirect index of tension applied to muscle.

In a previous study of shear elastic modulus, Akiyama et al. reported that shear elastic modulus of the lateral gastrocnemius (LG), medial gastrocnemius (MG), SOL, peroneus longus (PL), and tibialis anterior was significantly higher in MTSS patients who had pain at the time of measurement than in healthy subjects [14]. However, because the increase in muscle stiffness can be caused by muscle contraction due to the pain-induced spasms [15], the pain at the time of measurement may influence the shear elastic modulus. Moreover, a review article investigating the factors of MTSS occurrence reported that MTSS history was one of the strongest determinate risk factors [5]. Therefore, Saeki et al. investigated the effect of MTSS history on shear elastic modulus of the posterior lower leg, and reported that shear elastic moduli of the FDL and tibialis posterior (TP) in subjects with MTSS history were higher than in those without MTSS history [16]. The results of Saeki et al. suggest that increased shear elastic moduli of FDL and TP could be a mechanism of MTSS occurrence. Therefore, these previous studies suggested that higher shear elastic modulus of the posterior leg could be one of the reasons for the occurrence of MTSS.

However, previous studies have investigated shear elastic modulus in only MTSS patients who experienced pain at the time of measurement or in those who had a history of MTSS; it remains unclear whether the changes in the shear elastic modulus occurred after prolonged running, which is believed to cause MTSS. It is likely that a muscle whose shear elastic modulus increases after running may be related to MTSS. Therefore, the purpose of this study was to investigate the effect of 30 min of running on shear elastic modulus of the posterior lower leg in healthy subjects. We hypothesized that shear elastic moduli of the FDL and TP, which were higher in subjects with MTSS history than those without MTSS subject in previous study [16], would be increased after a 30-min running task.

## Methods

### Participants

Twenty healthy males volunteered to participate in this study (age, 20.9 ± 0.6 y; height, 169.6 ± 4.5 cm; weight, 62.6 ± 5.2 kg). We excluded subjects with a history of neuromuscular disease or musculoskeletal injury involving the lower extremities. None of the subjects were recreational or competitive runners; they were recreationally active but not involved in any ongoing strength training and stretching activities. All subjects were fully informed of the procedures and purpose of the study, and all provided written informed consent. This study was approved by the ethics committee at the Niigata University of Health and Welfare, Niigata, Japan.

### Experimental procedure

Before (PRE) and immediately after (POST) a 30-min running task, with a 4.5 min warm-up, the shear elastic modulus of the posterior lower leg in the dominant leg (right side for all subjects) was measured. The measurement details are described below.

### Assessment of shear elastic modulus of the of posterior lower leg

Subjects were seated in the Biodex System 4.0 (Biodex Medical Systems Inc., USA) at 90° of hip flexion. In addition, the dominant knee was maintained at full extension, and the ipsilateral foot was securely attached to the dynamometer footplate. The footplate being perpendicular to the fibula was defined as 0° dorsiflexion of the ankle joint. Subjects were instructed to remain relaxed during measurement.

**Fig. 1:**
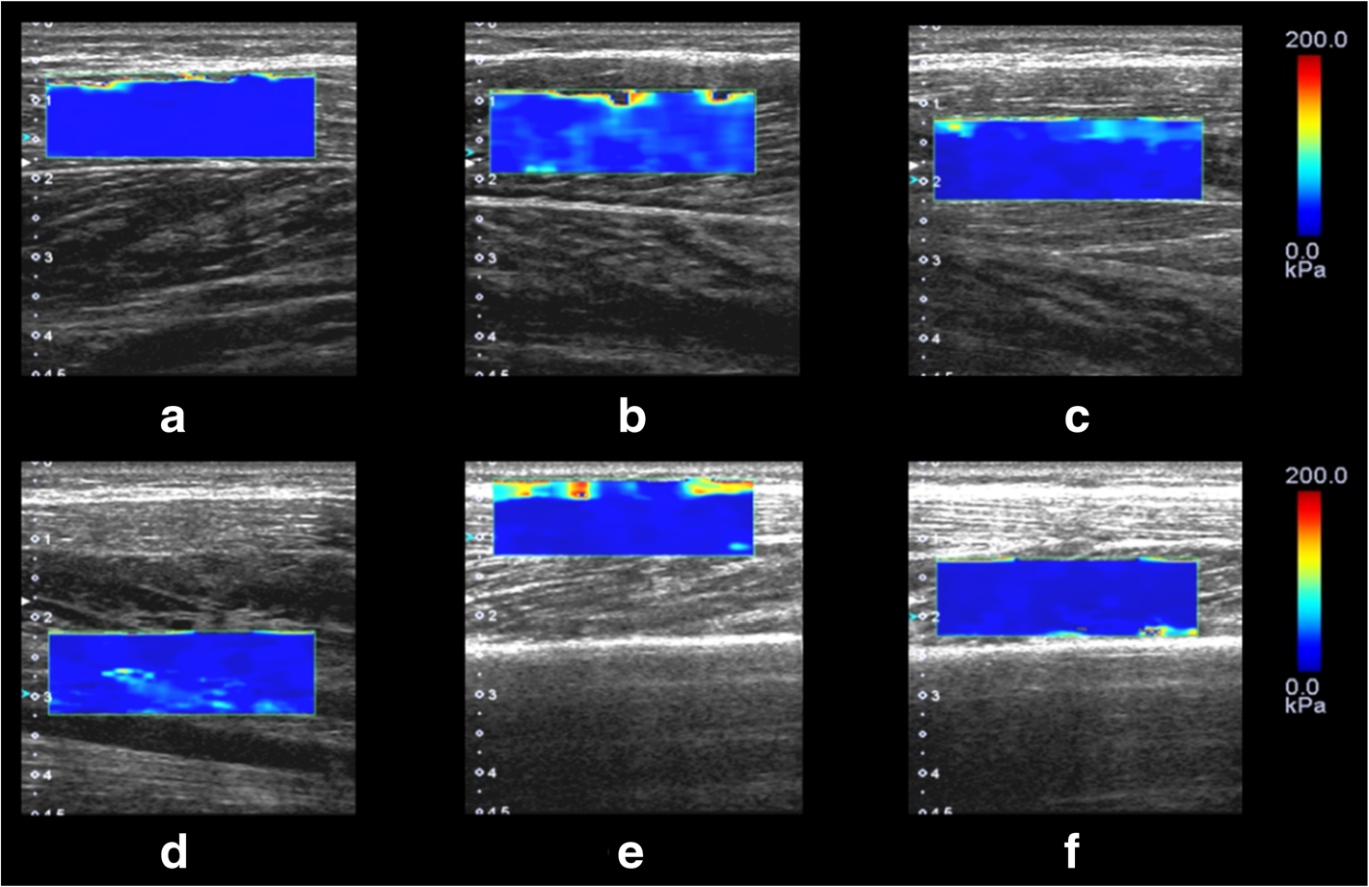
Elastographic ultrasound images of shear elastic modulus measurements. **a** Lateral gastrocnemius muscle, **b** Medial gastrocnemius muscle, **c** Flexor digitorum longus muscle, **d** Tibialis posterior muscle, **e** Peroneus longus muscle, **f** Peroneus brevis muscle

**Fig. 2:**
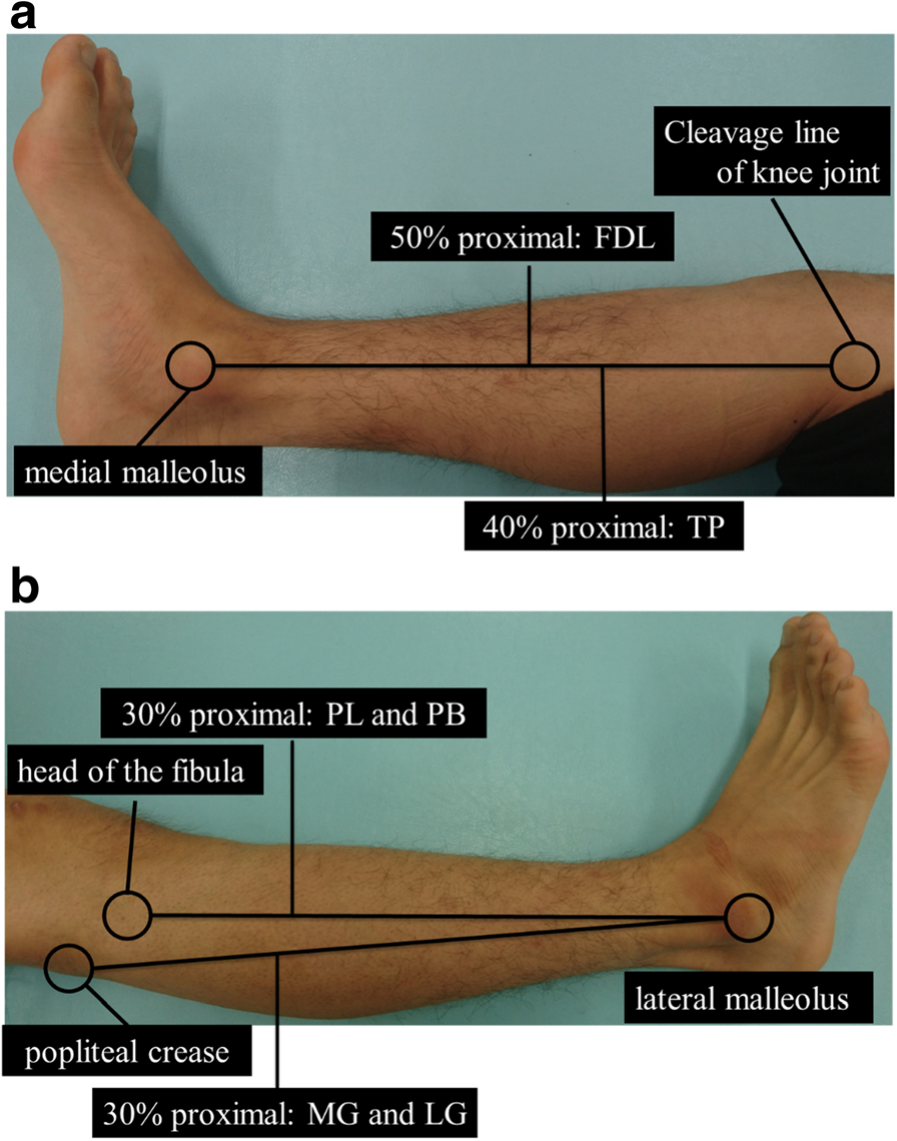
Locations of the shear elastic modulus measurement. **a** medial and **b** lateral sides. FDL: flexor digitorum longus; TP: tibialis posterior; LG: lateral gastrocnemius; MG: medial gastrocnemius; PL: peroneus longus; PB: peroneus brevis

The shear elastic modulus of the posterior lower leg was measured using ultrasonic SWE (Aplio 500, Toshiba Medical Systems, Tochigi, Japan) with a 5–14 MHz linear probe. As shown in Fig. 1, we measured the shear elastic moduli of the longitudinal axis of LG, MG, FDL, TP, PL, and peroneus brevis (PB) muscles. In addition, as shown in Fig. 2, the measurement locations were determined based on a previous study [16]. Briefly, shear elastic moduli of the LG and MG were measured at the proximal 30% of the lower leg length from the popliteal crease to the lateral malleolus. The shear elastic moduli of the PL and PB were measured at the proximal 30% from the head of the fibula to the lateral malleolus. The shear elastic modulus of the FDL was measured at the proximal 50% from the cleavage line of the knee joint to the medial malleolus. The shear elastic modulus of the TP was measured at the proximal 40% from the cleavage line of the knee joint to the medial malleolus for the superficial layer of the intramuscular tendon. After identification of the measurement location, we confirmed the measurement muscle by confirming movement of muscle fibers in a B-mode ultrasound image during passive movement of the ankle or toes. In addition, we marked the measurement site with an oil pen and ensured that measurements were recorded from the same site before and after running task.

In this study, the region of interest (ROI) was set near the center of each muscle. The obtained elastographic images were analyzed using image analysis software (MSI Analyzer version 5.0, Institute of Rehabilitation Science, Tokuyukai Medical Corporation, Japan). A quadrangular ROI was set to be as large as possible within the color-coded area of the elastographic images while accounting for artifact from aponeurosis, and the mean shear elastic modulus (kPa) in the quadrangular ROI was automatically calculated. The shear elastic modulus of each muscle was measured twice, and the average value was used for further analysis. Measurement of shear elastic modulus at POST was completed within 10 min after the running task.

### Running task

With reference to a previous study [17], we adopted the 30-min running task protocol, with a 4.5-min warm-up session to fatigue the lower-limbs. The subjects performed the required running test on a treadmill (AFR1016, ALINCO, Japan) set at 0° inclination for 34.5 min, including the 4.5-min warm-up, 1.5 min at 6 km/h, 1.5 min at 8 km/h, 1.5 min at 10 km/h, and 30 min at 12 km/h.

### Intersession reliability measurements of shear elastic modulus

To determine intersession measurement reliability of the shear elastic modulus, nine healthy males participated (age 21.0 ± 0.0 y; height 170.8 ± 7.4 cm; weight 68.5 ± 7.6 kg). The measurement protocol described above was performed twice by the same investigator with a 35-min rest period, which was approximately the same duration as the running task.

### Statistical analysis

Statistical analyses were performed using SPSS (version 21.0, SPSS Japan INC., Tokyo, Japan). The intersession measurement reliability was assessed using the intraclass correlation coefficient (ICC) (1, 1). The minimal detectable difference at 95% confidence interval (MDD_95%_) was calculated as follows: MDD_95%_ = z × SEM × √2, where z = 1.96 and standard error of measurement (SEM) = SD√(1 − ICC) [18]. Differences between PRE and POST for shear elastic modulus of each muscle were assessed using the paired *t*-test. A *P*-value <0.05 was considered to indicate statistical significance.

## Results

### Intersession reliability measurements of shear elastic modulus

The ICC (1, 1) and MDD_95%_ of intersession measurements are shown in Table 1. The ICC (1, 1) ranged from 0.709 to 0.985 for the shear elastic moduli of each muscle. Based on previous studies [19, 20], an ICC value of <0.40 is generally considered poor reliability, 0.40–0.75 as moderate to good, and >0.75 as excellent reliability. In this study, the measurement of the shear elastic modulus showed moderate to excellent reliability, consistent with the results of a previous study [21].

**Table 1:** Inter-session reliability and MDD_95%_ of shear elastic modulus measurement

kPa			ICC (1, 1)	reliability	MDD_95%_
LG	4.9 ± 1.4	4.8 ± 1.4	0.938	excellent	1.0
MG	11.3 ± 3.3	11.3 ± 3.3	0.916	excellent	2.6
FDL	4.3 ± 3.7	4.2 ± 3.7	0.985	excellent	1.2
TP	3.8 ± 1.0	3.7 ± 1.0	0.852	excellent	1.0
PL	6.5 ± 5.2	5.5 ± 4.7	0.709	moderate to good	6.8
PB	4.8 ± 3.0	4.4 ± 2.9	0.733	moderate to good	3.9

*ICC* Intraclass correlation coefficients, *MDD*_*95%*_ minimal detectable difference at the 95% confidence interval, *LG* lateral gastrocnemius, *MG* medial gastrocnemius, *FDL* flexor digitorum longus, *TP* tibialis posterior, *PL* peroneus longus, *PB* peroneus brevis

### Changes in shear elastic modulus before and immediately after a 30-min running task

Changes in shear elastic modulus of each muscle between PRE and POST are shown in Table 2. Shear elastic moduli of the FDL and TP at POST were significantly higher than PRE (FDL; PRE: 4.0 ± 3.8, POST: 5.5 ± 6.2 kPa, *p* = 0.019, TP; PRE 3.5 ± 1.6, POST: 4.5 ± 2.5 kPa, *p* = 0.035, respectively). However, there were no significant changes in shear elastic moduli of the MG, LG, PL, or PB between PRE and POST.

**Table 2:** Changes in shear elastic modulus before and immediately after a 30-min running task

kPa	PRE	POST	*p*-value
LG	6.2 ± 3.8	4.6 ± 2.5	0.146
MG	9.0 ± 6.0	8.3 ± 5.7	0.636
FDL	4.0 ± 3.8	5.5 ± 6.2*	0.019
TP	3.5 ± 1.6	4.5 ± 2.5*	0.035
PL	7.0 ± 9.6	5.8 ± 6.6	0.189
PB	4.2 ± 5.1	4.1 ± 4.7	0.407

*PRE* before running task, *POST* immediately after running task, *LG* lateral gastrocnemius, *MG* medial gastrocnemius, *FDL* flexor digitorum longus, *TP* tibialis posterior, *PL* peroneus longus, *PB* peroneus brevis

*P < 0.05; significantly different from PRE

## Discussion

In this study, we aimed to investigate the effect of a 30-min running task on shear elastic modulus of the posterior lower leg in healthy subjects. The results of this study revealed that shear elastic moduli of the FDL and TP were increased after a 30-min running task, but there were no changes in shear elastic moduli of the MG, LG, PL, or PB. To the best of our knowledge, this is the first report to investigate the effect of running, which is believed to cause MTSS, on shear elastic modulus of the posterior lower leg.

In this study, shear elastic moduli of the FDL and TP at POST were significantly higher than at PRE. The result confirmed our hypothesis and supported previous reports that shear elastic moduli of the FDL and TP were significantly higher in subjects with a history of MTSS than in those without a history [16]. A previous study showed that there was a significant correlation between shear elastic modulus measured by SWE and passive tension [12, 13], and the shear elastic modulus could be used as an indirect index of tension applied to muscle. Therefore, the passive tension applied to FDL and TP may have increased after the running task. In addition, Bouche et al. reported that strain in the tibial fascia increased as tension on the TP, FDL, and SOL tendons increased using the fresh cadaver specimens [22]. Taken together, we assumed that the increases in passive tension applied to FDL and TP after the running task leads to an increase in the strain on the tibial fascia, resulting in the development of MTSS. Therefore, it was suggested that the increases in shear elastic moduli of FDL and TP after running could be a risk factor for running-related MTSS development. In the future, a prospective study investigating the relationship between the increases in shear elastic moduli of FDL and TP after the running task and MTSS development should be conducted.

In this study, shear elastic modulus of the TP was increased after a 30-min running task, but the TP was not attached to the margin of the tibia, which is commonly affected by MTSS symptoms [10]. Therefore, it was unclear whether an increased shear elastic modulus of the TP might cause MTSS. However, Saeki et al. reported that shear elastic modulus of the TP in subjects with an MTSS history was significantly higher than that in subjects without an MTSS history [16], which is consistent with the results of this study. A previous study [16] found that a higher shear elastic modulus of TP could lead to myofascial force transmission to FDL, which is attached to a site commonly affected by MTSS [10]. Therefore, an increase in the shear elastic modulus of TP after running may lead to an increase in the passive tension on FDL via myofascial force transmission, which may be associated with MTSS development.

The results of this study revealed that there were no significant changes in shear elastic moduli of the LG, MG, PL, or PB between PRE and POST running task. A previous study showed that there were no significant differences in the shear elastic moduli of these muscles between subjects with and without a history of MTSS and who did not have pain at the time of measurement [16]. However, this previous study employed a cross-sectional design and shear elastic modulus was measured only at rest, whereas we investigated the effect of running, which is believed to cause MTSS, on shear elastic modulus of the posterior lower leg to expand on the previous findings [16]. Taken together, these results indicate that the elongation stress and hypertonicity of LG, MG, PL, and PB may not be associated with MTSS development.

This study has some limitations. First, the researcher who analyzed the elastographic images was not blinded to the subjects and measurement period. Second, as this was a pilot experiment, it included some subjects for whom the shear elastic modulus of Sol could not be measured. In addition, because the reliability of the shear elastic moduli of Sol and FHL could not be guaranteed, we did not measure these. Third, in this study, we investigated only the acute effect of running on the shear elastic modulus of healthy subjects and not of runners and MTSS patients. Thus, further study is needed to investigate the retention time of changes in the shear elastic modulus as well as the effects of repeated running tasks on the shear elastic modulus of the posterior lower leg in runners and MTSS patients to elucidate the chronic mechanism underlying MTSS development.

## Conclusions

This study investigated the acute effect of a 30-min running task on shear elastic modulus of the posterior lower leg in healthy subjects. The results of this study revealed that shear elastic moduli of the FDL and TP increased after a 30-min running task.

## Abbreviations

FDL: Flexor digitorum longus
ICC: Intraclass correlation coefficients
LG: Lateral gastrocnemius
MG: Medial gastrocnemius
MTSS: Medial tibial stress syndrome
PB: Peroneus brevis
PL: Peroneus longus
SWE: Shear wave elastography
TP: Tibialis posterior
